# The abyss of research funding in Brazil

**DOI:** 10.17179/excli2024-8037

**Published:** 2024-12-05

**Authors:** Lucindo José Quintans-Júnior, Fábio Guedes Gomes

**Affiliations:** 1 Universidade Federal de Sergipe (UFS). São Cristóvão, Sergipe, CEP 49100-000, Brazil; 2 CEO of Fundação de Amparo à Pesquisa do Estado de Alagoas (FAPEAL), Maceió, Alagoas, CEP 57020-330, Brazil; 3 Universidade Federal de Alagoas (UFAL), Maceió, Alagoas, CEP 57072-970, Brazil

## ⁯⁯⁯⁯

Despite significant progress in recent years, Brazil's scientific landscape remains starkly uneven, with funding heavily concentrated in a few affluent regions. This imbalance stifles innovation and limits the nation's overall scientific potential. While countries like China have implemented targeted programs, such as the Excellent Young Scientists Fund (EYS), to boost the productivity and impact of early-career researchers, Brazil continues to rely on traditional funding models that predominantly support well-established centers (Li et al., 2024[3]). This approach not only hinders the emergence of disruptive research but also exacerbates regional inequalities, leaving vast areas of the country underfunded and scientifically marginalized. Addressing these asymmetries is crucial for fostering a more inclusive and dynamic research environment.

"The science is back" is a phrase often said by Brazilian government authorities and President Lula himself. Indeed, science in Brazil has begun to receive increased investment, with the federal government treating universities and research centers with renewed respect, fostering dialogue, and expanding internationalization opportunities through more assertive policies in this area. A positive indicator is the Financier of Studies and Projects (FINEP), which, through the National Fund for Scientific and Technological Development (FNDCT), allocates resources for research and infrastructure improvements. However, a historical wound persists: persistent regional asymmetry in resource distribution (Rodrigues, 2023[6]).

Despite statements from the Minister of Science, Technology, and Innovation (MCTI), Luciana Santos, advocating for better resource allocation to regions like the North and Northeast, where asymmetries are most pronounced, there is no well-established policy to address this imbalance (de Oliveira Andrade, 2024[1]). Although FINEP's budget includes calls to fund research in these regions and parts of the Midwest, actions to mitigate this problem are still insufficient. Funding continues to favor more developed regions, undermining efforts to reduce disparities and contradicting the promise of "science for all." This inconsistency between rhetoric and action is discouraging (Quintans-Júnior, 2024[4]).

Interestingly, the execution of FNDCT reveals a concentration of resources in the Southeast region (SE). In 2023, out of R$ 4.8 billion (US$ 0.9 billion) in non-reimbursable resources, 46 % went to defense, space, and aeronautics companies, with 82 % of this amount concentrated in the SE, of which 75 % was in São Paulo alone. Additionally, 25 % went to Science and Technology Institutions, with 55 % in the SE. Finally, 29 % financed strategies of MCTI and CNPq (Brazilian funding agency) (FINEP, 2023[2]).

In 2020, Quintans Júnior et al. (2020[5]) raised concerns about the increasing asymmetry and persistent underfunding of science in Brazil, emphasizing its potential to hinder long-term progress. At the time, the federal budget proposal (PLOA) projected a nearly 4 % reduction in funding for CAPES (Brazilian funding agency for graduate programs) and CNPq, the country's main agencies for research and higher education, compared to 2020. However, by the end of the year, the cuts surpassed 10 %, exacerbating an already critical situation. These reductions compounded a broader trend: between 2015 and 2021, university budgets were slashed by 29 %, and research funding declined by an alarming 41 %. Such drastic measures threaten the sustainability of scientific innovation, eroding Brazil's potential for global competitiveness and its ability to address pressing societal challenges.

Something that remains puzzling after the period of limited access to funding agencies during the Bolsonaro administration is that, despite the current availability of more resources and more robust funding programs, financial support continues to be concentrated in the southeastern and southern regions of Brazil. This leaves the other regions-characterized by greater asymmetries, poverty, and urgent needs-receiving minimal funding. These areas require significantly higher investments to become competitive and to meet the very demands defined by the Brazilian government as essential for the country's development.

The FNDCT Deliberative Council should reexamine the allocation and utilization of its resources, implementing policies and issuing calls that enhance research infrastructure in underdeveloped regions, thereby bolstering the foundation for innovation and entrepreneurship. China's strategic investment in science, such as the EYS Fund, has shown that targeted funding can effectively cultivate research capabilities and reduce regional disparities. Brazil must adopt similarly assertive measures to ensure that equitable resource distribution is not just a rhetorical commitment but a tangible pathway to inclusive scientific progress. Otherwise, the vision of balanced development will remain an unfulfilled promise. 

## Declaration

### Funding

None. 

### Acknowledgments

LJQJr is research productivity (PQ) fellows at the National Council for Scientific and Technological Development (CNPq), Brazil.

### Conflict of interest

The authors declare no conflict of interest.
